# Validation of suitable house keeping genes for hypoxia-cultured human chondrocytes

**DOI:** 10.1186/1471-2199-10-94

**Published:** 2009-10-09

**Authors:** Casper Bindzus Foldager, Samir Munir, Michael Ulrik-Vinther, Kjeld Søballe, Cody Bünger, Martin Lind

**Affiliations:** 1 Orthopaedic Research Laboratory, Aarhus University Hospital, Aarhus, Denmark; 2 Sports Trauma Clinic, Aarhus University Hospital, Aarhus, Denmark

## Abstract

**Background:**

Hypoxic culturing of chondrocytes is gaining increasing interest in cartilage research. Culturing of chondrocytes under low oxygen tension has shown several advantages, among them increased synthesis of extracellular matrix and increased redifferentiation of dedifferentiated chondrocytes. Quantitative gene expression analyses such as quantitative real-time PCR (qRT-PCR) are powerful tools in the investigation of underlying mechanisms of cell behavior and are used routinely for differentiation and phenotype assays. However, the genes used for normalization in normoxic cell-cultures might not be suitable in the hypoxic environment. The objective of this study was to determine hypoxia-stable housekeeping genes (HKG) for quantitative real-time PCR (qRT-PCR) in human chondrocytes cultured in 21%, 5% and 1% oxygen by geNorm and NormFinder analyses.

**Results:**

The chondrocytic response to the hypoxic challange was validated by a significant increase in expression of the hypoxia-inducible gene ankyrin repeat 37 as well as SOX9 in hypoxia. When cultured on the 3-dimentional (3D) scaffold TATA-binding protein (TBP) exhibited the highest expression stability with NormFinder while Ribosomal protein L13a (RPL13A) and beta2-microglobulin (B2M) were the most stable using geNorm analysis. In monolayer RPL13A were the most stable gene using NormFinder, while geNorm assessed RPL13A and human RNA polymerase II (RPII) as most stable. When examining the combination of (3D) culturing and monolayer RPL13A and B2M showed the highest expression stability from geNorm analysis while RPL13A also showed the highest expression stability using NormFinder. Often used HKG such as beta actin (ACTB) and glyceraldehyde-3-phosphate dehydrogenase (GAPDH) were the most unstable genes investigated in all comparisons. The pairwise variations for the two most stable HKG in each group were all below the cut-off value of 0.15, suggesting that the two most stable HKG from geNorm analysis would be sufficient for qRT-PCR.

**Conclusion:**

All data combined we recommend RPL13A, B2M and RPII as the best choice for qRT-PCR analyses when comparing normoxic and hypoxic cultured human chondrocytes although other genes might also be suitable. However, the matching of HKG to target genes by means of a thorough investigation of the stability in each study would always be preferable.

## Background

Hypoxic culturing of chondrocytes is gaining increasing interest in cartilage research. Culturing of chondrocytes under low oxygen tension has shown several advantages, among them increased synthesis of extracellular matrix and increased redifferentiation of dedifferentiated chondrocytes [1,2]. Studying this particular cell type in low oxygen concentrations is of interest in terms of mimicking the in vivo environment and investigating the characteristics of chondrocytes exposed to these factors [3].

Gene expression analyses are powerful tools in the investigation of underlying mechanisms of cell behavior and are used routinely not only for differentiation and phenotype assays, but also for quantification of gene expression. Although there are other approaches for quantification, this is often performed by the use of quantitative real-time reverse transcriptase polymerase chain reaction (qRT-PCR), in which expression magnitude is measured in correlation to the expression of a reference gene [4]. The reference gene is selected specifically for its properties of exhibiting stable expression under such diverse and changing stimuli as growth factors and oxygen tension in the culture environment. These genes are often referred to as housekeeping genes (HKG) because they are necessary for cell survival and thus, their syntheses are present in all nucleated cell types. Despite the obvious importance of the stability of HKG, normalization is one of RT-PCRs most difficult tasks [5], and the selection of HKG when used in a specific setup is often either only briefly discussed or its importance more or less overlooked.

Several studies have shown that a HKG that is appropriate in one study setup for a given cell type might not be suitable in another setup [6]. Furthermore, recently published studies have shown that often-used HKG are unsuitable for qRT-PCR [6-8]. These traditional HKG are often genes coding for structural cell compartments such as β-actin. However, it is known that for some cells the cytoskeleton is modulated during culturing and also if cultured on different surfaces. In addition, metabolic pathway genes such as glyceraldehyde-3-phosphate dehydrogenase (GAPDH) or 18s ribosomal RNA (18S) are used. This may be explained partly by the fact that HKG are not only implicated in the basal cell metabolism, but also participate in other cell functions [9,10], and were already between 1975 and 1987 described as being regulated under certain conditions [11-13]. For this reason it has been proposed at least two HKG should be used [6,14]. Previously, Zhong et al. have pointed out that significant changes in expression levels of commonly used HKG are exhibited under hypoxia in four different cell lines [15]. In vivo, chondrocytes are normally exposed to low oxygen tension, and thus the gene expression response to hypoxia might not reflect that of other cell types [16].

Many studies concerning hypoxia-cultured chondrocytes do not describe the background for selecting HKG for quantification, and in comparison between hypoxic- and normoxic cultured chondrocytes, no consistency in the use of HKG exists [1,17-23]. Different scaffolds for matrix-assisted chondrocyte implantation (MACI) treatments are also being investigated in vitro. The effect of three-dimensional culturing of chondrocytes on candidate HKG variability is not clarified. We hypothesize that low oxygen tension affects the expression levels of genes usually considered stable in normoxic conditions in human chondrocytes cultures. Due to the importance of appropriate HKG selection and the increased use of hypoxia culturing for cartilage research, the aim of this study is to reveal HKG exhibiting stable expression in both hypoxic- and normoxic environment and over time in both monolayer cultures and on 3-dimensional (3D) scaffolds by means of a direct comparison.

## Methods

### Cartilage samples

Cartilage biopsies were collected from the intercondylar groove in the distal femur from 12 healthy patients undergoing anterior cruciate ligament reconstruction. The biopsies were collected after obtaining the patients' written consent and the protocol was approved by the local ethical committee under the Danish National Committee on Research Ethics. The biopsies were transported to the laboratory facility in a suspension of DMEM/F-12 with Glutamax (Gibco-Invitrogen), 10% FCS (Invitrogen), streptomycin and penicillin (Sigma-Aldrich) and Gentamicin (Sigma-Aldrich). Each biopsy was cut into smaller pieces followed by digestion using 0.1% collagenase II (Gibco) and 0.1% hyaloronidase (Sigma-Aldrich) for 18-20 hours at 37°C in a waterbath. The cells were then washed in DMEM/F-12 (Gibco-Invitrogen) and seeded in a 10-cm^2^-culture dish in standard culture media containing 10% FCS and the antibiotics mentioned above.

### Scaffolds

Methoxypolyethyleneglycol-block-co-poly(lactide-co-glycolide (MPEG-PLGA) (ASEED, Coloplast A/S, Humlebæk, Denmark) (50:50 LA:GA) was dissolved on 100 ml 1,4 dioxane overnight at 50°C. Seven milliliter of polymer solution was poured into a precooled aluminum mold 7.3 × 7.3 cm^2 ^and placed in a freeze dryer at -20°C and 1 hour at + 30°C. The scaffolds were then dried overnight in a vacuum dessicator at room temperature. The scaffolds were sterilized in 100% EtOH, dried and packed into aluminized PET-bags. The average porosity of the scaffolds was above 90% and the thickness 2.0 mm. These scaffolds have previously been used in MACI treatment in an animal model [24].

### Cell culture

Isolated chondrocytes were cultured separately for each patient in DMEM/F12 medium with antibiotics in normoxia (21% oxygen tension) until they reached confluence. Following trypsination (1.25% trypsin and 5 mM EDTA), the cells were divided to monolayer or scaffold seeding. For the monolayer culture, cells were seeded in 24-well plates with a density of 20,000 cells/cm^2 ^i.e. 38,000 cells/well. MPEG-PLGA scaffolds soaked in culture medium (4 mm in diameter) were placed in agarose coated 24-well plates to prevent cells from adhering to the wells. 125.000 cells in 10 μL media were added on top of the wet scaffold (seeding concentration 5 × 10^6 ^cells/mL). The scaffolds were left in an incubator for 1.5 hours to allow cells to adhere, followed by gentle addition of 1 mL culture medium. Twenty four hours after seeding, the cells for baseline investigation (t = 0) were harvested. The remainders were divided into three groups for incubation in either normoxia (21% oxygen tension), hypoxia (5% oxygen tension) or severe hypoxia (1% oxygen tension) in a designated hypoxia workstation (Xvivo System, BioSpherix, NY) that was pre-balanced for the desired growth environment. RNA extractions from these subcultures were performed after 1, 2 and 6 days after the baseline time-point respectively (passage 2). Oxygen and carbon dioxide tensions as well as temperature were measured throughout the experiment.

### Total RNA extraction

Scaffolds with cells were vortexed in 1 mL TRIzol^® ^Reagent (Invitrogen, Taastrup, Denmark) and total RNA was extracted according to the manufacturer's instructions and dissolved in nuclease-free water (Ambion, Cambridgeshire, UK). Total RNA from monolayer cells was extracted with the GenElute Mammalian Total RNA Miniprep Kit (Sigma-Aldrich). Finally, the concentration of RNA was spectrophotometrically assessed by means of the Quant-iT™ RiboGreen^® ^RNA Kit (Molecular Probes) according to manufacturer's instructions. Each sample was measured in a 96-well plate using a microplate reader. RNA quality analysis was performed using an Agilent 2100 Bioanalyzer (Agilent Technologies, Santa Clara, CA). All measurements were performed according to the manufactures' instructions.

### qRT-PCR

The RNA samples were treated with DNase I (Ambion, Cambridgeshire, UK) and converted into complementary DNA (cDNA) using the High Capacity cDNA Archive Kit (Applied Biosystems, Naerum, Denmark). Real-time quantitative polymerase chain reaction (qRT-PCR) was performed on a 7500 Fast Real-Time PCR system (Applied Biosystems, Naerum, Denmark) using commercially available TaqMan^® ^Gene Expression Assays (Applied Biosystems) Glyceraldehyde-3-phosphate dehydrogenase (GAPDH) Hs99999905_m1, Ubiquitin C (UBC) Hs00824723_m1, 18S rRNA (18S) Hs99999901_s1, Ribosomal protein L13a (RPL13A) Hs03043885_g1, TATA-binding protein (TBP) Hs00427621_m1, β_2_-microglobulin (B2M) Hs99999907_m1, β-actin (ACTB) Hs99999903_m1, hypoxanthine phosphoribosyltransferase 1 (HPRT1) Hs99999909_m1and a Custom TaqMan^® ^Gene Expression Assay for human RNA polymerase II (RPII). Forward primer sequence of RPII was GACACAGGACCACTCATGAAGT and reverse primer sequence was GTGCGGCTGCTTCCATAAG. Standard enzyme and cycling conditions for the 7500 Fast System were used. Amplicon size was <180 bp for all primer sets to maximize the amplification efficiency. Cell response to hypoxic challenge was confirmed by means of gene expression analysis of the hypoxia-inducible gene ankyrin repeat domain 37 (ANKRD37) as described by Benita et al. [25] and SOX9 expression [26] using TaqMan^® ^Gene Expression Assays (Applied Biosystems) Hs00699180_m1 and Hs00165814_m1 respectively.

Template cDNA corresponding to 4 ng of RNA was added to each PCR reaction and each biological sample was run in technical duplicates for each gene. Data analysis was performed using 7500 Fast System Sequence Detection Software version 1.3 (Applied Biosystems, Naerum, Denmark).

### Data analysis and statistics

Data analysis was performed using geNorm [14] and NormFinder [27] software packages. The geNorm algorithm relies on the principle that the expression ratio of two ideal HKG is identical in all samples regardless of the experimental condition. The program applies a statistical algorithm to calculate the average stability measure (M) of each candidate gene as the average pairwise variation (V) for that gene to the control genes. The HKG are then ranked by stepwise exclusion of the gene with the highest M. The genes with the lowest M are considered most stable. V is used to determine the optimal number of HKG, as more than one might be needed. V_n/(n+1) _was calculated to determine the possible need to include more than two HKG for normalization. As originally described by Vandesompele et al. [14], the cut-off value was set to 0.15, below which the inclusion of an additional HKG is not required. NormFinder generates a similar measure of stability - a lower value implies a higher stability in gene expression using a model-based approach as described by Andersen et al. [27].

A two-tailed T-test was used to reveal any significant differences in the normalized SOX9 and ANKRD37 expression. P-values less than 0.05 were considered significant.

## Results and Discussion

RNA quality analyses showed RNA Integrity Values (RIN) of 7.2-9.8 (median 9.0). The algorithm assigns an RIN number score from 1 to 10, where level 10 represents completely intact RNA and 1 represents a highly degraded RNA. Thus, a sufficient RNA quality was obtained in this study.

### Hypoxic response

**Figure 1:**
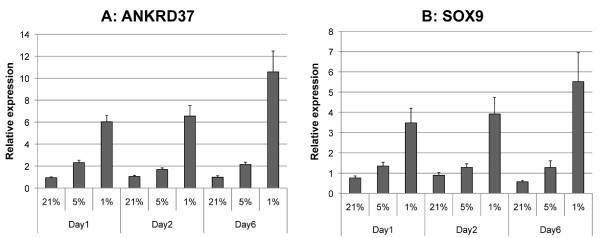
**Expression of ANKRD37 (A) SOX9 (B)**. Relative mean expression is presented on day 1, 2, and 6 in human chondrocytes cultured in 21%, 5% or 1% oxygen (error bars: SEM). RPL13A and B2M are used as reference genes. Values for each sample are normalized to baseline expression.

Investigation of normalized expression showed that lowering oxygen tension significantly increased expression of ANKRD37 between the three oxygen tensions at all time-points (P < 0.01) (figure 1A). SOX9 expression showed a significant increase with lowering of oxygen tension to 1% (P < 0.05). However, the increase was not significant between 21% and 5% (figure 1B). Thus, a hypoxic challenge to the cells was successfully achieved and the cells remained viable with lowered oxygen tension.

### Monolayer

**Figure 2:**
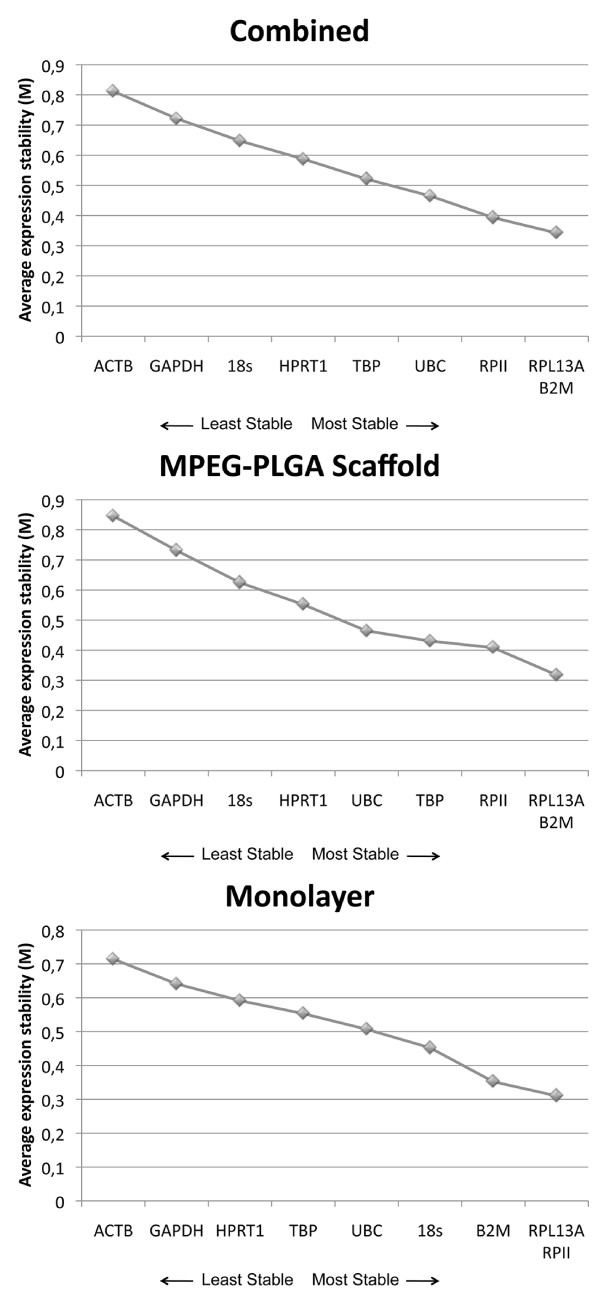
**Average expression stability measure (M) of candidate HKG**. During stepwise exclusion of the least stable gene in monolayer, 3D culturing in MPEG-PLGA scaffolds, and the combined stability for all samples M is calculated.

**Figure 3:**
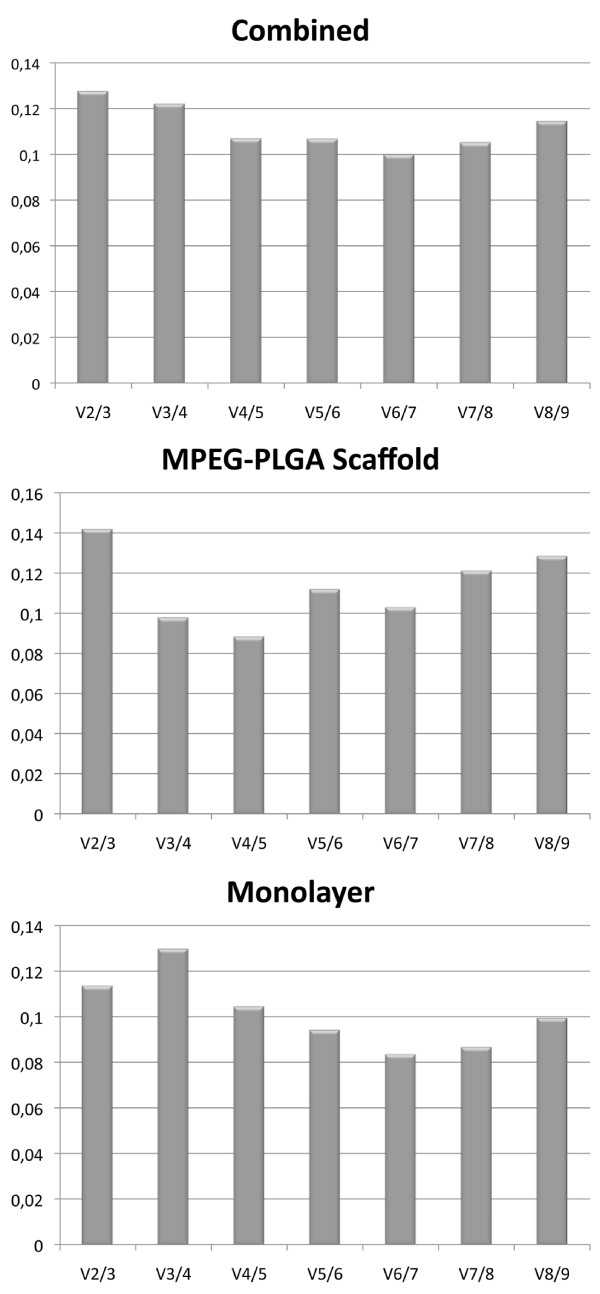
**Pairwise variation (V)**. V between two sequential normalization factors containing an increasing number of genes in monolayer, 3D culturing in MPEG-PLGA scaffolds, and the combined stability for all samples is calculated.

NormFinder assessed RPL13A as the most stable gene (table 1). Using geNorm analysis, we ranked the nine candidate HKG regarding the average expression stability (M) as follows: ATCB > GAPDH > HPRT1 > TBP > UBC > 18S > B2M > RPL13A and RPII, the latter being the most stable (figure 2). Pairwise variation (V) showed V2/3 of 0.129, which is below the cut-off value of 0.15 (figure 3). Thus, RPL13A and RPII would be sufficient for qRT-PCR analysis.

**Table 1:** NormFinder stability values.

**Gene name**	**Stability Value**		
	**Monolayer**	**Scaffold**	**Combined**
RPL13A	0.255 (1)	0.246 (2)	0.284 (1)
RPII	0.296 (5)	0.322 (4)	0.309 (2)
HPRT1	0.292 (2)	0.364 (5)	0.328 (3)
TBP	0.377 (7)	0.243 (1)	0.333 (4)
UBC	0.280 (2)	0.385 (7)	0.338 (5)
B2M	0.357 (6)	0.286 (3)	0.345 (6)
18S	0.294 (4)	0.374 (6)	0.389 (7)
GAPDH	0.399 (8)	0.643 (8)	0.534 (8)
ACTB	0.607 (9)	0.788 (9)	0.701 (9)

Lowest value equals highest stability. Bracket numbers shows the ranking of the stability in the three groups.

### Scaffold

NormFinder assessed TBP as the most stable gene (table 1). Using geNorm analysis, we ranked the nine candidate HKG regarding the average expression stability (M) as follows: ATCB > GAPDH > 18S > HPRT1 > UBC > TBP > RPII > RPL13A and B2M, the latter being the most stable (figure 2). Pairwise variation (V) showed V2/3 of 0.141, which is below the cut-off value of 0.15 (figure 3). Thus, the use RPL13A and B2M would be sufficient for qRT-PCR analysis.

### Combined data

NormFinder assessed RPL13A as the most stable gene (table 1). Using geNorm analysis, we ranked the nine candidate HKG regarding the average expression stability (M) as follows: ATCB > GAPDH > 18S > HPRT1 > TBP > UBC > RPII > RPL13A and B2M, the latter being the most stable (figure 2). Pairwise variation (V) showed V2/3 of 0.127, which is below the cut-off value of 0.15 (figure 3). Thus, the use RPL13A and B2M would be sufficient for qRT-PCR analysis.

### Interpretation

The findings in this study clearly illustrate the differences in the stability of HKG in human chondrocytes cultured in hypoxia and normoxia. A previously performed study on arthritic cartilage showed that TBP, RPL13A and B2M were suitable for use as HKG. However, that study did not reflect expression levels of in vitro cultured chondrocytes, since the biopsies were frozen immediately after collection for subsequent thawing and investigation as organ culture. Moreover, it is hard to conclude the environmental conditions of the cells, since the cartilage was exposed to 21% oxygen tension for up to 6 hours before freezing, which is a considerable exposure in terms of possible changes in gene expression patterns. In our study, the lysis of the cells was performed in the hypoxic workstation before the subsequent steps, thus preventing changes in gene expression.

RPL13A encodes a ribosomal protein that is a component of the 60S subunit in the ribosome and belongs to the L13P family of ribosomal proteins located in the cytoplasm. The gene has previously been shown to be a reliable HKG in different cell types, but has not yet been described for hypoxic use [28-31]. However, in this study it was the most stable HKG of all investigated candidates. RPL13A has several known processed pseudogenes, which can obscure quantification of the RPL13A gene. This must be taken into consideration when designing the primer. However, the commercially available primer used in this study has a design that is specific to the target gene and hence avoids detection of pseudogenes.

B2M is part of the MHC class I molecule, which is present on almost all cells. Despite the previous lack of evidence in the literature for hypoxic applications, it has been used in several studies on hypoxia-culturing chondrocytes [20,21,32]. However, in this study we have shown that B2M is a reliable HKG when comparing hypoxic and normoxic cultured chondrocytes. Although RPII presented a stable expression in this study, it is not a commonly used HKG. However, it has previously been shown to be a suitable reference gene for human mesenchymal stem cells [33,34], while it has shown to be ineffective as HKG for qRT-PCR in bovine myocytes [35].

TBP has previously been described as a suitable reference gene for chondrogenic differentiation of adipose-derived stem cells and is also suggested as HKG in osteoarthritic cartilage [8,28]. In this study, TBP expressed high stability in both geNorm and NormFinder analyses.

UBC has been shown to be suitable as a reference gene for only a few cell types such as colon cancer cells [27], porcine oocytes [36]. Otherwise the use of UBC is limited. It shows only relatively stable expression in monolayer culture in the NormFinder analysis and is therefore, based on the findings of this study not recommended as a general chondrocytic HKG.

Although often used, GAPDH has in several cell types revealed its ineffectiveness as HKG due to its lack of stability. One part of the explanation might be due to induced transcription of GAPDH by HIF-1α, which increases dramatically under low oxygen tension [37]. It has moreover proven to be affected in developing cells [38]. Despite these controversies and the issues demonstrated in this study, the use of this gene for normalization has been reported in hypoxia culturing of chondrocytes [22,23].

Both analytical tools disclosed that ACTB was the most unstable gene in both monolayer and 3D culturing. Despite previous findings of ACTB as an unsuitable HKG, it has recently been used in an otherwise interesting study concerning increased matrix synthesis in chondrocyte cultures exposed to sustained hypoxic environment [1]. Based on this study we cannot recommend the use of this HKG as a normalization factor in qRT-PCR. In general ACTB and GAPDH had the most unstable expression found by both geNorm and NormFinder analyses and based on this study the use of these genes as HKG should be avoided when comparing hypoxic and normoxic cultured chondrocytes.

18S is a cleavage product of the 45S gene repeats and is often used as HKG in chondrocyte studies as well as in several other cell studies [39,40]. However, several precautions must be taken into consideration when this gene is used. Firstly, it is important to be aware that 18S is ribosomal RNA (rRNA), while the often-pursued target genes are investigated as mRNA. rRNA and mRNA differ in the kinetic of the synthesis and breakdown due to several different factors such as secondary structure (protein folding), sequence, and function. Secondly, 18S has a low Ct-value (i.e. high expression) compared both to other potential HKG and to many target genes. Furthermore, rRNA fraction may count for up to about 80% of the RNA in a sample [4]. When using more than one HKG, the lack of stability of 18S might result in a technical issue inasmuch as the high expression does not allow the qRT-PCR software to produce a reliable measurement of baseline amounts of RNA for this HKG.

Many studies concerning hypoxia-cultured chondrocytes do not describe the background for selecting HKG for quantification, and comparisons of hypoxic and normoxic cultured chondrocytes show no consistency in the use of HKG. In studies investigating hypoxic culturing of chondrocytes, even fewer HKG are selected based on calculation and relationship to the target genes [17]. Furthermore, there is no tradition for using more than one HKG in qRT-PCR evaluations [21]. In qRT-PCR studies the selection of HKG is a crucial step towards obtaining reliable results. Previously, Bas et al. [41] and Tricarico et al. [42] have shown that wrong reference genes can lead to alterations in the results, which emphasizes that more than a single HKG should be used in order to increase the stability. When the opportunity of investigating suitable HKG for a specific setup is not present, it is highly recommended that the previously published literature be extensively studied and that more than one reference gene be used. To our knowledge, there are unfortunately no previous publications on HKG selection for hypoxia-cultured chondrocytes.

When selecting a series of multiple HKG in a study, the functionality of the assay (primer and probe) should be considered. Due to the properties of the assay used for 18S-detection, this might also detect genomic DNA when present, while the assay for RPL13A most certainly will detect genomic DNA. DNase-treating was therefore necessary before conversion into cDNA. If the target gene can be detected with an assay complement only to mRNA, DNase-treatment would be a redundant step. Due to the opportunity of skipping the DNase-step, selection of a HKG with an appropriate mRNA detecting assay might be preferable.

GeNorm analysis revealed that pairwise variation (V) values for V2/3 was below the cut-off value of 0.15 in all scenarios. Thus, the use of two HKG would be sufficient for a stable and valid reference in qRT-PCR. Based on this study we recommend the use of at least two of the following genes: RPII, B2M, RPL13A. In parallel, the study from Pombo-Suarez et al. [28] concerning osteoarthritic cartilage in elderly patients found B2M, RPL13A and TBP to be the best HKG candidates.

The most obvious finding from the NormFinder analysis was the unstable expression of ACTB and GAPDH across all experimental conditions. The variations expression of these genes were much larger than the rest of the candidate HKG, which again underline their ineffectiveness as HKG. The stability value of the candidate HKG, with the exception of ACTB and GAPDH, did not differ very much using NormFinder. The stability values were all between 0.243 and 0.389, which according to this analytical tool indicates that they all might be useful as HKG. However, when combined with the geNorm analysis it becomes evident that RPL13A, B2M, and RPII have a higher stability in both monolayer- and scaffold-culturing. It should be noticed that if the target genes have an intergroup gene variability greater than the HKG variation, this HKG might be sufficient in order to obtain reliable results [4].

This study investigated the comparison of three different oxygen tensions in human chondrocytes cultured in either monolayer or on a 3D scaffold and therefore our recommendations concern these experimental conditions only.

## Conclusion

We recommend B2M, RPII and RPL13A as the best choice for qRT-PCR analyses of normoxic and hypoxic cultured human chondrocytes although other genes might also be suitable. However, the matching of HKG to target genes by means of a thorough investigation of the stability in each study would always be preferable.

## Authors' contributions

CF, ML and SM participated in the protocol writing, the analyses, and the drafting of the manuscript. KS, CB and MU participated in the design of the study protocol and performed the application to the ethical committee. CF carried out the study. All authors read and approved the final manuscript.
